# Association Between Pulse Pressure and Carotid Intima-Media Thickness Among Low-Income Adults Aged 45 Years and Older: A Population-Based Cross-Sectional Study in Rural China

**DOI:** 10.3389/fcvm.2020.547365

**Published:** 2020-11-12

**Authors:** Jie Liu, Qiuxing Lin, Dandan Guo, Yuan Yang, Xin Zhang, Jun Tu, Xianjia Ning, Yijun Song, Jinghua Wang

**Affiliations:** ^1^Department of Neurology, Tianjin Medical University General Hospital, Tianjin, China; ^2^Laboratory of Epidemiology, Tianjin Neurological Institute, Tianjin, China; ^3^Tianjin Neurological Institute, Key Laboratory of Post-Neuroinjury Neuro-Repair and Regeneration in Central Nervous System, Ministry of Education and Tianjin City, Tianjin, China; ^4^Department of General Medicine, Tianjin Medical University General Hospital, Tianjin Neurological Institute, Tianjin, China

**Keywords:** pulse pressure, carotid intima-media thickness, risk factors, epidemiology, atherosclerosis

## Abstract

Worldwide, the stroke burden remains severe, especially for people in low socioeconomic groups. Atherosclerosis is a leading cause of stroke that is attracting increasingly greater attention. Blood pressure, including pulse pressure (PP) and systolic (SBP) and diastolic (DBP) blood pressures, is a traditional risk factor for atherosclerosis; its association with carotid intima-media thickness (CIMT) has also been widely studied. However, published studies have not reported on the relationship between PP and CIMT in low-income adults. Thus, this study investigated the relationship between PP and CIMT in a low-income population, in China. A total of 3,789 people, aged ≥45 years and without histories of stroke or cardiovascular disease, were recruited into this study. B-mode ultrasonography was performed to determine CIMTs. Demographic characteristics, physical examination data, previous medical histories, and laboratory test results were collected for each study participant. Multiple linear regression models were used to analyze the association between CIMT and PP. The mean CIMT was 567.1 μm (males, 583.5 μm; females, 555.7 μm). The SBP, DBP, PP, and mean arterial pressure (MAP) values were all positively correlated with CIMT, in the univariate analysis; PP and MAP showed the strongest correlations. In addition, in three multiple linear regression models, PP was shown to be significantly associated with CIMT; each 1-mm Hg increase in PP resulted in a CIMT increase of ≥0.41 μm (all *P* < 0.001). Our results demonstrated that, when compared with SBP, DBP, and MAP, PP may be the best predictor of CIMT. Thus, controlling blood pressure, especially PP levels, is vital to decreasing the prevalence of atherosclerosis, especially in this low socioeconomic status population in China.

## Introduction

Stroke is the main cause of death and disability, worldwide (1), and is the leading cause of death in China (2). Each year, in China, the absolute costs of medical treatments for stroke surpass those reported for the United States in 2013; annually, China is estimated to spend $7.87 billion on stroke-related medical treatment, mostly for post-stroke care and rehabilitation (3). As the leading cause of stroke, atherosclerosis causes 10–20% of cerebral infarction events (4) and 20–30% of ischemic stroke events (5)Carotid intima-media thickness (CIMT) is a useful measure for detecting early atherosclerotic changes (6), and previous studies have demonstrated that an increased CIMT is a marker of atherosclerosis (7, 8). Thus, early detection of CIMT risk factors and their early modification may significantly impact the prevention of atherosclerotic disease.

Blood pressure is known to play a major role in the occurrence and progression of atherosclerosis. In the Young Finns cohort, adolescent systolic blood pressure (SBP) was shown to predict CIMT in adulthood (9). In women with coronary artery disease, followed for 3.2 years, high baseline pulse pressures (PPs) were associated with coronary atherosclerosis progression (10). In fact, studies have shown a direct association between PP and CIMT (11, 12). A previous study of adults with hypertension demonstrated that diastolic blood pressure (DBP) was lower in the group with increased CIMTs than it was in the group with normal CIMTs; PPs and the PP index [(SBP-DBP)/DBP] were significantly higher in the group with increased CIMTs (13).

Presently, China is a developing country and its low-income population accounts for a large proportion of the national population. Because of their lower incomes and poorer medical care, people in this socioeconomic class have greater risks of stroke and cardiovascular disease; however, they are unable to access timely medical assistance. To our knowledge, few other studies have examined this relationship, especially among low-income populations with low levels of educational attainment and living in rural areas of China. This study, therefore, investigated the association between PP and CIMT in such a population.

## Methods

### Participants and Study Design

This cross-sectional survey included 18 villages in rural areas of Tianjin, China, and involved participants from the previously described Tianjin Brain Study (14–16), conducted between April 2014 and January 2015. In short, about 95% of the total area population (14,251 people) were low-income farmers, with 2014 per capita disposable incomes of <1,600 USD (17). In 2011, the average length of formal education for the population was 5.26 years. From this population, 3,789 residents (≥45 years old) were recruited into this study. Individuals with previous histories of or current symptomatic cardiovascular (including myocardial infarctions, angina, and asymptomatic myocardial ischemia) or cerebrovascular (including ischemic and hemorrhagic strokes) diseases were excluded.

The surveys were approved by the ethics committee of Tianjin Medical University General Hospital. The study was conducted according to approved guidelines and written informed consent was obtained from each participant.

### Risk Factors and Physical Examinations

This study involved face-to-face interviews conducted by trained researchers. The name, sex, date of birth, education level, individual and family medical histories [including the presence or occurrence of hypertension, stroke, diabetes mellitus (DM), transient ischemic attacks, and coronary heart disease], and lifestyle factors (including cigarette smoking, defined as smoking ≥1 cigarette/day for ≥1 year; passive smoking, defined as ≥1 person smoking in the room; and alcohol consumption, defined as drinking ≥500 g of alcohol/week for ≥1 year).

Each participant underwent a physical examination to measure blood pressure, height, and weight; the body mass index (BMI) was calculated by dividing the weight (kg) by the square of height (m). Fasting blood glucose (FBG), triglyceride (TG), total cholesterol (TC), low-density lipoprotein cholesterol (LDL-C), and high-density lipoprotein cholesterol (HDL-C) levels were measured. Carotid ultrasound examinations were also conducted.

### Ultrasonography Measurements

All ultrasound examinations and measurements were performed by one trained technician who was blinded to participant details. Each patient was placed in a supine position, and was examined using B-mode ultrasonography (Terason 3000; Burlington, Massachusetts, MA, US) with a 5–12-MHz linear array transducer. Two extracranial carotid trees (i.e., the common carotid artery, bifurcation, internal carotid artery, and the internal and external carotid arteries) were measured to obtain the CIMT; the vessels were also screened for the presence of plaque. CIMTs of the proximal and distal walls of the common carotid artery were measured on the left and right sides, and the maximum, minimum, and average CIMT values were obtained. Carotid plaques were defined as local structural invasions into the arterial lumen of at least 0.5 mm or 50% of the surrounding CIMT value, or when the distance between the intima-lumen interface and the media adventitia interface was >1.5 mm (18); the longitudinal and transverse dynamic images of each plaque were digitally stored, according to the standard protocol (19). Regardless of the measured carotid plaque values, participants with carotid plaques were included in the carotid plaque group if >1 lesion was found. The ranges of inter- and intra-observer correlation coefficients for the CIMT measurements were 0.88–0.94 and 0.80–0.95, respectively.

### Survey Procedure

Local village doctors visited all eligible residents, in accordance with pre-determined procedures, 1 day before the physical examination. Between April 15, 2014 and June 30, 2014, the physical examinations (including blood pressure, weight, height, carotid ultrasound, and 12-lead echocardiography) and blood sample collections were performed in the local clinics. All blood samples were sent to the Central Laboratory of Tianjin Medical University General Hospital for determination of TC, TG, HDL-C, and LDL-C levels within 12 h of collection; FBG levels were determined at the Central Laboratory of Tianjin Ji County People's Hospital within 2 h of sample collection. Between July 1, 2014 and January 8, 2015, carotid plaque and CIMT measurements were determined by one practiced technician.

### Definitions of Risk Factors

Mean arterial pressure (MAP) was calculated as (SBP + 2 × DBP)/3 and PP was calculated as SBP – DBP. Hypertension was defined as SBP ≥ 140 mmHg and/or DBP ≥ 90 mmHg (20). DM was defined as a FBG concentration ≥7.0 mmol/L. Obesity was defined as a BMI ≥ 30.0 kg/m^2^ and overweight was defined as a BMI of 25.0–29.9 kg/m^2^.

### Statistical Analyses

Continuous variables are reported as means and standard deviations; Student's *t*-test was used to analyze between-group differences in TC, TG, HDL-C, and LDL-C levels. Categorical variables are expressed as frequencies and 95% confidence intervals (CIs) and between-group comparisons were made using chi-square tests. The participants were divided into four age groups (45–54, 55–64, 65–74, and ≥75 years old) and into three educational groups, based on the length of formal education (0, 1–6, and ≥7 years). Logistic regression analyses were used to evaluate the CIMT risk factors in both males and females.

The univariate analysis results are shown as unadjusted B-values and 95% CIs; the multivariate analysis results are shown as adjusted B-values and 95% CIs, after adjusting for covariates. The age (45–54 years old) and educational (≥7 years) groups were analyzed as categorical variables; hypertension, DM, obesity, current smoking, passive smoking, drinking, high TC, high TG, low HDL-C, and high LDL-C levels were analyzed as dichotomous variables. P-values of statistically significant were considered to be <0.05 for the dichotomous variables, *P* < 0.0166 for three groups of categorical variables, and *P* < 0.0125 for four groups of categorical variables.

A linear regression model was used to examine the association of PP and MAP with CIMT. Three models were developed. Model 1 involved adjusting for age (continuous variable), sex (male or female), and education (continuous variable). Model 2 included the Model 1 variables plus hypertension (categorical variable), DM (categorical variable), smoking (categorical variable), and drinking (categorical variable); Model 3 included the Model 2 variables plus FBG, TG, HDL-C, LDL-C, and the HDL-C/LDL-C ratio (all continuous variables). SPSS for Windows (version 19.0; SPSS, Chicago, IL, USA) was used for the analyses.

## Results

### Participant Characteristics

This study recruited 3,789 individuals, aged ≥45 years (mean age, 59.92 years), including 1,560 (41.2%) men and 2,229 (58.8%) women. For these participants, the average length of formal education was 5.48 years, and 17.4% of the participants had never received any formal education (8.8% of men and 23.4% of women); however, 37.9% of the participants had received formal educations lasting ≥6 years. The average SBP and DBP levels were high in this population, with mean values of 146.42 mmHg and 86.81 mmHg, respectively. The mean PP and MAP values were 59.61 mmHg and 106.68 mmHg, respectively (Table 1).

**Table 1:** Demographic characteristics and risk factors for all participants.

**Risk factors**	**Women**	**Men**	**Total**
Cases, *n* (%):	1,560 (41.2)	2,229 (58.8)	3,789 (100)
Age, mean (SD)	59.07 (9.47)	61.13 (9.90)	59.92 (9.70)
**Age group**, ***n*** **(%)**			
45–54 years	806 (36.2)	430 (27.6)	1,236 (32.6)
55–64 years	882 (39.6)	632 (40.5)	1,514 (40.0)
65–74 years	386 (17.3)	338 (21.7)	724 (19.1)
≥75 years	155 (7.0)	160 (10.3)	315 (8.3)
**Education**, ***n*** **(%)**			
0 years	522 (23.4)	137 (8.8)	659 (17.4)
1–6 years	995 (44.6)	699 (44.8)	1,694 (44.7)
> 6 year	712 (31.9)	724 (46.4)	1,436 (37.9)
**Smoking status**, ***n*** **(%)**			
Never smoking	2,099 (94.2)	743 (47.6)	2,842 (75.0)
Ever smoking	27 (1.2)	146 (9.4)	173 (4.6)
Current smoking	103 (4.6)	671 (43.0)	774 (20.4)
**Alcohol consumption**, ***n*** **(%)**			
Never drinking	2,199 (98.7)	999 (64.0)	3,198 (84.4)
Ever drinking	1 (0)	48 (3.1)	49 (1.3)
Current drinking	29 (1.3)	513 (32.9)	542 (14.3)
Hypertension, n (%):	1,472 (66.0)	1,111 (71.2)	2,583 (68.2)
Diabetes, n (%):	317 (14.5)	216 (14.1)	533 (14.3)
**BMI**, ***n*** **(%)**			
Normal	912 (41.8)	738 (48.1)	1,650 (44.4)
Overweight	950 (43.6)	626 (40.8)	1,576 (42.4)
Obese	282 (12.9)	145 (9.5)	427 (11.5)
SBP, mean (SD)	145.49 (22.64)	147.76 (21.41)	146.42 (22.17)
DBP, mean (SD)	85.62 (11.39)	88.50 (11.22)	86.81 (11.40)
PP, mean (SD)	59.86 (17.51)	59.26 (17.03)	59.61 (17.32)
MAP, mean (SD)	105.58 (13.75)	108.25 (13.12)	106.68 (13.56)
FBG, mean (SD)	5.93 (1.67)	5.91 (1.42)	5.92 (1.57)
TC, mean (SD)	5.04 (1.11)	4.62 (1.10)	4.87 (1.09)
TG, mean (SD)	1.87 (1.22)	1.61 (1.24)	1.76 (1.24)
HDL-C, mean (SD)	1.50 (4.76)	1.39 (0.43)	1.46 (0.46)
LDL-C, mean (SD)	2.76 (1.28)	2.61 (1.20)	2.70 (1.25)
HDL-C/LDL-C, mean (SD)	0.88 (1.65)	0.74 (0.94)	0.83 (1.41)

*SD, standard deviation; BMI, body mass index; SBP, systolic blood pressure; DBP, diastolic blood pressure; PP, pulse pressure; MAP, mean arterial pressure; FBG, fasting blood glucose; TC, total cholesterol; TG, triglycerides; HDL-C, high-density lipoprotein cholesterol; LDL-C; low-density lipoprotein cholesterol*.

### Mean CIMT Differences Grouped by Demographic Characteristics and Risk Factors

Table 2 shows the mean CIMT values for the participants, grouped by demographic characteristics and risk factors. The mean CIMT increased from 536.3 μm (45–54-year-old group) to 609.0 μm (≥75-year-old group). However, the mean CIMT decreased as the group education level increased. The mean CIMT was significantly higher in current smokers than in never smokers, and it was also higher among alcohol consumers than among those who did not consume alcohol (*P* < 0.001). Compared with the non-hypertensive population, patients with hypertension had a significantly higher mean CIMT (*P* < 0.001). Similarly, the mean CIMT for patients with DM was higher than that for patients without DM (*P* < 0.001).

**Table 2:** Differences in mean carotid intima-media thicknesses, according to demographic characteristics and risk factors groups.

**Risk factors**	**Mean (SD) (μm)**	***t*/*F***	***P***
Sex:		9.487	<0.001
Males	583.5 (92.64)		
Females	555.7 (82.59)		
Age group:		103.534	<0.001
45–54 years	536.3 (75.91)		
55–64 years	571.4 (85.30)		
65–74 years	592.6 (93.26)		
≥75 years	609.0 (91.85)		
Education:		36.368	<0.001
0 years	583.7 (94.02)		
1–6 years	573.2 (87.91)		
>6 year	552.4 (82.76)		
Smoking status:		24.665	<0.001
Never smoking	555.7 (90.34)		
Ever smoking	574.8 (80.60)		
Current smoking	580.5 (94.73)		
Alcohol consumption:		15.965	<0.001
Never drinking	563.7 (85.40)		
Ever drinking	597.5 (89.29)		
Current drinking	584.5 (99.32)		
Hypertension:		13.055	<0.001
Yes	579.1 (89.19)		
No	541.5 (79.30)		
Diabetes:		3.812	<0.001
Yes	580.4 (85.61)		
No	564.7 (88.21)		
BMI:		1.155	0.326
Normal	565.2 (88.94)		
Overweight	569.2 (86.79)		
Obese	566.9 (83.96)		

*BMI, body mass index*.

### Associations Between the Mean CIMT and Measured Parameters in the Linear Regression Analysis

A linear regression analysis showed that age, PP, MAP, SBP, DBP, FBG, TC, and LDL-C were positively correlated with CIMTs, but education, TG, and HDL-C levels, and the HDL-C/LDL-C ratio were negatively correlated with CIMTs (all, *P* < 0.05). Each 1-mmHg increase in PP or MAP was associated with mean CIMT increases of 1.16 μm (95% CI, 1.00–1.32; *P* < 0.001) and 1.02 μm (95% CI, 0.82–1.23; *P* < 0.001), respectively. Moreover, SBP, DBP, FBG, TC, and LDL-C were positively associated with CIMT in the univariate analysis (β = 0.85, 0.56, 4.65, 3.40, and 6.57, respectively; all *P* < 0.05; Table 3).

**Table 3:** Association of mean carotid intima-media thickness with measured parameters in the linear regression analysis.

**Parameters**	**β**	**SE**	**95% CI**	***P***
PP	1.16	0.08	(1.00, 1.32)	<0.001
MAP	1.02	0.10	(0.82, 1.23)	<0.001
Age	2.52	0.14	(2.24, 2.80)	<0.001
Education	−3.08	0.40	(−3.86, −2.29)	<0.001
SBP	0.85	0.63	(0.73, 0.98)	<0.001
DBP	0.56	0.12	(0.31, 0.80)	<0.001
BMI	0.22	0.39	(−0.94, 0.98)	0.568
FBG	4.65	0.92	(2.85, 6.44)	<0.001
TC	3.40	1.32	(0.80, 5.99)	0.010
TG	−2.97	1.16	(−5.25, −0.69)	0.011
HDL-C	−6.51	3.13	(−12.65, −0.38)	0.037
LDL-C	6.57	1.15	(4.32, 8.82)	<0.001
HDL-C/LDL-C	−4.59	1.02	(−6.59, −2.58)	<0.001

*β, regression coefficient; SE, standard error; CI, confidence interval; SBP, systolic blood pressure; DBP, diastolic blood pressure; FBG, fasting blood glucose; TC, total cholesterol; TG, triglycerides; HDL-C, high-density lipoprotein cholesterol; LDL-C, low-density lipoprotein cholesterol*.

### Associations Between the Mean CIMT and the PP and MAP in the Multivariate Analysis

The associations between the mean CIMT and the PP and MAP, in the multivariate analysis, are shown in Table 4. The association between CIMT and PP remained statistically significant in Model 1. Moreover, PP was an independent risk factor for increased CIMT in Models 2 and 3; each 1-mmHg increase in PP was associated with CIMT increases of 0.42 μm (95% CI, 0.20–0.63; *P* < 0.001) and 0.41 μm (95% CI, 0.18–0.63; *P* < 0.001), respectively. However, there was no significant association between MAP and CIMT in Models 2 and 3 (both, *P* > 0.05).

**Table 4:** Association of mean carotid intima-media thickness (μm) with pulse pressure, according to demographic characteristics and risk factor groups in the multivariate analysis.

**Effect factors**	**Model 1 β (95% CI)**	**Model 2 β (95% CI)**	**Model 3 β (95% CI)**
PP	0.51 (0.30, 0.72)^*^	0.42 (0.20, 0.63)^*^	0.41 (0.18, 0.63)^*^
MAP	0.37 (0.13, 0.61)^*^	0.01 (−0.27, 0.30)	0.01 (−0.28, −0.30)

*Model 1 analysis adjusted by age, sex, and education; Model 2 analysis adjusted by age, sex, education, hypertension, diabetes, smoking, and drinking; Model 3 analysis adjusted by age, sex, education, hypertension, diabetes, smoking, drinking, fasting blood glucose; triglycerides; high-density lipoprotein cholesterol (HDL-C); low-density lipoprotein cholesterol (LDL-C); HDL-C/LDL-C. 95% CI, 95% confidence interval; PP, pulse pressure; MAP, mean arterial pressure*.

* *represents P < 0.05*.

## Discussion

To our knowledge, this was the first population-based study to explore the relationship between PP and CIMT in a low socioeconomic status study population. All of the included participants were low-income residents of the target area, were ≥45 years old, and had low educational attainment. The results of the multivariate analysis showed that PP was an independent risk factor for increased CIMT. Each 1-mmHg increase in PP was associated with a CIMT increase of at least 0.41 μm. However, there was no association between MAP and CIMT, after adjusting for covariates.

Although the relationship between blood pressure and CIMT has been demonstrated in many studies, few studies have explored the relationship between PP and CIMT, especially in a low socioeconomic status population. PP, a significant independent risk factor for cardiovascular mortality (21), has been shown in some experimental and clinical studies to modulate CIMT (13, 22, 23). After a 4-year follow-up, the Etude du Vieillissement Artériel study demonstrated that elevated PP levels were associated with CIMT progression, and increased CIMT was associated with PP widening (12). The Muscatine Offspring Study reported that, in adolescents, the CIMT increased by 0.232 mm for each 1-mmHg increase in PP (22). A Belgian study reported that each 1-mmHg increase in the 24-h PP resulted in a 139-μm CIMT increase (18). A study from France showed that, after adjusting for confounding variables, an increased CIMT was the only factor significantly and independently associated with high PP, irrespective of the effectiveness of blood pressure control and antihypertensive drug treatments (19). Our previous study showed a positive association between SBP and CIMT and a negative association between DBP and CIMT (16). However, the current study showed different results in the univariate analysis. In view of the collinearity between PP and SBP or DBP, the present study assessed the relationship between CIMT and PP by adjusting for other covariables, including hypertension, using a multivariate analysis. As a result, the current study showed that PP was an independent risk factor for CIMT in this low-income population in China. A continuously elevated blood pressure is well-known to impose a great mechanical burden on the vascular endothelium; subsequently, vascular structures and function gradually become impaired, ultimately resulting in vascular fibrosis, stiffness, and decreased intrinsic compliance (24). In turn, vascular stiffness and decreased compliance are often associated with the early stages of atherosclerosis, when CIMT first increases (25, 26). Pressure pulsation may directly affect atherosclerosis through a variety of mechanisms (27). For example, high pulsation can induce endothelial dysfunction (28), while cyclical strain can enhance monocyte adhesion to endothelial cells and regulate gene expression in smooth muscle cells and monocytes/macrophages (29–31).

As shown in previous studies, the trend toward higher CIMTs in individuals with only primary educations, or less, was more significant than in individuals with secondary or high school educations (32). More than 40% of China's population live in rural areas (17); these people tend to have poor health insurance, low educational attainment, and low incomes (33). Although the prevalence of hypertension in rural adults is similar to that for adults living in urban areas, the detection and medical treatment of hypertension in rural-living adults remains unsatisfactory (34). The unusually large urban-rural gap in hypertension treatment and the expanding trend toward hypertension detection require health policymakers and researchers to pay more attention to rural populations.

There are several limitations in this study. First, with respect to the study design, an inherent limitation of a cross-sectional study is that it cannot determine causal links between significant variables and CIMT; thus, additional longitudinal studies are needed to determine causality. Secondly, the reliance on low-income, poorly educated rural residents in China may limit the study's generalizability. However, this study population may represent other low socioeconomic status populations. Additionally, we did not analyze information regarding medication use among the participants; however, the frequency of medication use is low in this low socioeconomic status population. Thus, the absence of this information may not have had a significant impact on the validity of the present results. Finally, the study did not include a high-income comparator group. Further studies are needed to explore the differences between low- and high-income populations.

## Conclusions

This study focused on the relationship between PP and CIMT in low socioeconomic status adults in China. When compared with SBP, DBP, and MAP, PP appears to be the best predictor of CIMT. CIMTs were associated with increasing PP levels, and each 1-mmHg increase in PP resulted in a CIMT increase of at least 0.41 μm, in this low socioeconomic population. These findings suggest the need to both control blood pressure levels and monitor PP levels to decrease the prevalence of atherosclerosis and reduce the burden of cardiovascular disease and stroke, especially among this low-socioeconomic status population, in China.

## Data Availability Statement

The raw data supporting the conclusions of this article will be made available by the authors, without undue reservation.

## Ethics Statement

The studies involving human participants were reviewed and approved by the ethics committee of Tianjin Medical University General Hospital. The patients/participants provided their written informed consent to participate in this study.

## Author Contributions

JW, YS, and XN were involved in conception and design and data interpretation for this article. JW was involved in data analysis for this article. JL, QL, and DG was involved in manuscript drafting. JL, QL, DG, YY, XZ, and JT were involved in data collection, case diagnosis and confirmation for this article. JW, YS, and XN were involved critical review for this article. All authors contributed to the article and approved the submitted version.

## Conflict of Interest

The authors declare that the research was conducted in the absence of any commercial or financial relationships that could be construed as a potential conflict of interest.
